# Oncogenic Forkhead box D3 antisense RNA 1 promotes cell survival and confers temozolomide resistance in glioblastoma cells through the miR-128-3p/WEE1 G2 checkpoint kinase axis

**DOI:** 10.1080/21655979.2022.2042133

**Published:** 2022-02-22

**Authors:** Zaisheng Ling, Jinpeng Zhang, Qingqing Liu

**Affiliations:** a Department of Ct Diagnosis, The Second Affiliated Hospital of Harbin Medical University, Harbin, P. R. China; b Department of Rehabilitation, The Second Affiliated Hospital of Heilongjiang University of Chinese Medicine, Harbin, P. R. China; c Department of Neurology, The First Affiliated Hospital of Harbin Medical University, Harbin, P. R. China

**Keywords:** ceRNA, FOXD3-AS1, glioblastoma (GBM), miR-128-3p, WEE1

## Abstract

Although temozolomide (TMZ) is recommended for glioblastoma (GBM) treatment, patients treated with TMZ usually develop TMZ resistance. Thus, there is an urgent need to elucidate the mechanism through which GBM cells acquire TMZ resistance. FOXD3-AS1, a recently discovered lncRNA, shows high expression in diverse cancer types. Nonetheless, its role in GBM remains unclear. This study found that FOXD3-AS1 was overexpressed in GBM cells and associated with dismal prognostic outcome in GBM patients. Functional studies revealed that depletion of FOXD3-AS1 inhibited cell growth and induced apoptosis of GBM cells. Results also showed that FOXD3-AS1 participates in the tolerance of GBM cells to TMZ. Specifically, TMZ-resistant cells exhibited higher FOXD3-AS1 expression compared to parental cells. Overexpression of FOXD3-AS1 increased TMZ tolerance in TMZ sensitive cells, whereas depletion of FOXD3-AS1 sensitized TMZ-resistant cells to TMZ treatment. Mechanistically, WEE1 was positively expressed with FOXD3-AS1. Given that both FOXD3-AS1 and WEE1 contain a binding site for miR-128-3p, FOXD3-AS1 could act as a competing endogenous RNA (ceRNA) to promote WEE1 expression by sponging miR-128-3p. Furthermore, we demonstrated that WEE1 was upregulated in TMZ-resistant GBM cells. Overexpression of WEE1 increased TMZ tolerance in TMZ sensitive cells, whereas deletion of FOXD3-AS1 promoted TMZ-resistant cells to be more sensitive to TMZ. Importantly, depletion of WEE1 could reverse TMZ resistant phenotype in FOXD3-AS1-overexpressed GBM cells. Collectively, our findings reveal a critical role of FOXD3-AS1 in the survival of GBM cells and TMZ resistance, which suggests that FOXD3-AS1 is a potential biomarker for the diagnosis and treatment of GBM.

## Introduction

Glioblastoma (GBM) is a primary brain malignancy with high mortality [1]. GBM patients usually have a poor prognosis, and a large percentage of GBM cases suffer recurrence and develop drug resistance [2,3]. Several studies have proved that the inherence of great heterogeneities in tumor facilitates GBM relapse and chemoresistance [4,5].

Over the years, temozolomide (TMZ) has been extensively utilized as a chemotherapy to treat GBM [6]. Notably, TMZ is a DNA-alkylating agent, where the methyl group is added at O^6^ position on the guanine of genomic DNA [7]. Studies have shown that TMZ promotes G2/M arrest in the cell cycle, ultimately resulting in apoptosis [8]. However, over 50% of GBM patients develop TMZ resistance and thus cannot benefit from TMZ treatment. Therefore, this calls for studies to elucidate the molecular mechanisms underlying TMZ resistance in GBM and identify novel targets to reverse TMZ resistance.

Long non-coding RNAs (lncRNA) are RNA molecules with over 200 nucleotides, but less of protein coding potential [9]. Numerous studies have suggested that lncRNAs regulate gene expression through different mechanisms, such as acting as ceRNAs of miRNA [10] and directly binding to functional proteins [11]. Additional evidences have revealed that lncRNAs participate in different human diseases, especially in regulating chemoresistance in human cancer [12]. For instance, lncRNA MIR22HG regulates GBM progression through Wnt/β-catenin signaling [13], whereas HOTAIRM1 promotes tumor aggressiveness and radiotherapy resistance in glioblastoma [14]. One study also found that targeting lncRNA MALAT1 by a nanocomplex with siRNA sensitizes glioblastoma to TMZ [15].

Forkhead box D3 antisense RNA 1 (FOXD3-AS1) is a novel lncRNA involved in multiple cancers. A previous study demonstrated that FOXD3-AS1 regulates the miR-325/MAP3K2 axis to promote the development of malignant skin melanoma [16]. Besides, FOXD3-AS1 expression is associated with the clinical development of breast cancer (BC), and modulates BC cell invasion and migration [17]. With exception of one study that found that FOXD3-AS1 suppresses the development of non-small cell lung cancer (NSCLC) [18], almost all studies have suggested that FOXD3-AS1 plays an oncogenic role. However, its function in GBM remains unclear.

Herein, we hypothesized that lncRNA FOXD3-AS1 could promote the survival of GBM cells and induce TMZ-resistance. The main aim of the study was to explore the role of FOXD3-AS1, and elucidate its underlying molecular mechanism in the survival of GBM cells and TMZ-resistance in GBM. Results demonstrated that the upregulation of FOXD3-AS1 in GBM cells was correlated with poor prognosis. Notably, FOXD3-AS1 upregulation was also detected in TMZ-resistant GBM cells. On the other hand, downregulation of FOXD3-AS1 inhibited cell growth and induced apoptosis of GBM cells. Moreover, overexpression of FOXD3-AS1 increased TMZ tolerance in TMZ-sensitive GBM cells, whereas downregulation of FOXD3-AS1 promoted the TMZ sensitivity of TMZ-resistant cells. Mechanistically, FOXD3-AS1 could be the ceRNA that upregulates WEE1 through the miR-128-3p sponge. FOXD3-AS could also contribute to TMZ resistance through promoting WEE1 G2 checkpoint kinase (WEE1) expression. Collectively, our results revealed an important role of FOXD3-AS1 in cell survival and TMZ resistance in GBM cells, which suggests that FOXD3-AS1 may serve as a prognostic biomarker candidate and a potential therapeutic target for GBM treatment.

## Methods and reagents

### Cell culture

Human GBM cell lines, including A172, U87, U251, LN118, and T98, were provided by Cell Bank of the Chinese Academy of Sciences (Shanghai, China), whereas the healthy human NHA astrocytes were provided by Lonza (Basel, Switzerland). All cells were cultured in RPMI1640 media (Invitrogen, Carlsbad, CA, USA) supplemented with 1% penicillin–streptomycin (Invitrogen) and 10% fetal bovine serum (FBS; Invitrogen), and incubated in a humid incubator under 5% CO_2_ and 37°C conditions.

To generate stable TMZ resistant U87 cells, parental U87 cells were treated with TMZ for six months at gradient doses of 10–150 μM. The resistant U87 cells were then dispersed into single-cell layers within the 96-well plates, followed by incubation with TMZ (150 μM) to obtain TMZ-resistant subclones referred to as U87-R cells.

### Cell transfection

The FOXD3-AS1 siRNA sequence (siRNA#1: GAUGCUGGGAUGUGGAUUU, siRNA#2: CUCCAAGAUUUAACUUCCA), WEE1 siRNA sequence (AAUAGAACAUCUCGACUUA), and negative control siRNA sequence (CGUACGCGGAAUACUUCGAUU) were synthesized by Sangon, Shanghai, China. MiR-128-3p mimics and NC mimics were purchased from GenePharma, Shanghai, China, whereas FOXD3-AS1 and WEE1 expression vectors were obtained from GeneCopoeia, Guangzhou, China.

Briefly, U87 and U87-R cells were inoculated into 6-cm dishes 24 h before transfection. After cells adhered to the dishes, lipofectamine 2000 (Invitrogen) was used to transfect the above-indicated sequences into cells using serum-free medium. After 5 h, cells were subcultured into the serum-containing medium. Finally, cell lysates were collected 48 h post-transfection.

### RNA extraction and real-time quantitative polymerase chain reaction (qRT-PCR)

After cell lysis, TRIzol reagent (Invitrogen) was used to extract total cellular RNA. RNA concentrations were measured using a NanoDrop. Next, 1 μg total RNA was reverse transcribed to cDNA using a superscript II kit (Invitrogen) in accordance with the manufacturer’s instructions. The Power SYBR Green PCR Master Mix (Life Technologies, USA) was then adopted for the qRT-PCR procedure. The PCR conditions comprised 30 cycles, including 20s under 94°C, 20s under 60°C, and 30s under 72°C. Relative mRNA expression was calculated using the 2^−ΔΔCt^ approach.

The following primer sets were used to measure FOXD3-AS1 expression:

FOXD3-AS1-F: 5’- GGTGGAGGAGGCGAGGATG −3’;

FOXD3-AS1-R: 5’- GGTGGAGGAGGCGAGGATG −3’

β-actin-F: 5’- CATGTACGTTGCTATCCAGGC −3’;

β-actin-R: 5’- CTCCTTAATGTCACGCACGAT −3’.

β-actin was used for normalization.

The following primer sets were used to determine miR-128-3p expression:

miR-128-3p-F: 5’- GGGTCACAGTGAACCGGTC −3’

miR-128-3p-R: 5’- ATTGCGTGTCGTGGAGTCG −3’

U6-F: 5’- GCTTCGGCAGCACATATACTAAAAT −3’

U6-R: 5’- CGCTTCACGAATTTGCGTGTCAT −3’

U6 served as the reference for normalization.

### Protein isolation and Western blot analysis

Cells were lysed with protease inhibitor cocktail-containing RIPA buffer (Roche, Switzerland), and the protein concentration was measured by the Bradford method (Bio-Rad, Hercules, CA, USA). Next, equal protein volumes were resolved using SDS-PAGE, followed by transfer onto nitrocellulose membranes. The membranes were then blocked using 5% skimmed milk powder, followed by overnight incubation with primary antibodies against Actin (Santa Cruz, USA) nad PCNA, CDK4, CDK6, and cleaved caspase-1 (Cell signaling technology, USA) at 4°C. On the next day, membranes were rinsed with PBST and incubated with the corresponding mouse or rabbit secondary antibody (Cell signaling, USA) for 1 hour at room temperature. Finally, an ECL reagent (GE healthcare, USA) was used to visualize the blots.

### MTT assay

Cell viability was measured using the MTT assay [19]. Briefly, the transfected U87 and U87-R cells (4000/well) were inoculated in 96-well plates and cultured for six days. At day 0, day 3, and day 6, cells were stained with 20 μl of the 5 mg/ml MTT dissolved in PBS (Sigma, St. Louis, MO, USA) for 4 h at 37°C. After careful aspiration, 150 μl of dimethyl sulfoxide (DMSO) were added to each well, followed by measuring the absorbance (OD) value at 490 nm.

### Colony formation assay

Cell survival was measured using the colony formation assay [20]. After transfection with specific reagents for 24 h, 500 colonies of U87 cells or U87-R cells were counted and seeded into 35 mm dishes, and then cultured for 14 days. Next, colonies were stained for 20 min using 0.1% crystal violet dye containing 20% methanol. Finally, the clone number was determined and images captured.

### Acridine Orange/ethidium bromide (AO/EB) fluorescence staining

Cell apoptosis was measured by AO/EB staining [20]. Briefly, U87 or U87-R cells were transfected with the indicated reagents for 48 h and then incubated with the AO/EB solution for 5 min (Solarbio Biotechnology, China). Fluorescence microscopy was conducted to evaluate changes in cell morphology under 200 × . The apoptotic cell proportion was then determined as follows: apoptosis rate (%) = apoptotic cell number/total cell count *100%.

### TUNEL assay

TUNEL assay was applied to measure the cell apoptosis [21]. Briefly, U87 cells or U87-R cells were transfected with the indicated reagents for 48 h and then they were fixed with 4% PFA for a period of 15 min. Next, cells were permeabilized with the 0.25% Triton X-100 under ambient temperature for a period of 10 min. After rinsing, cells were incubated for 10 min with TdT reaction buffer, followed by incubation with Click-iT reaction cocktail for 30 min under ambient temperature. Subsequently, cells were washed with PBS, and DAPI was utilized to counter-stain cell nuclei. Stained coverslips were mounted with prolong diamond antifade mountant (Applied Biosystems, USA), and images were captured using a fluorescence microscope.

### RNA binding protein immunoprecipitation (RIP) assay

RIP assay was conducted by employing the EZ-Magna RIP RNA-Binding Protein Immunoprecipitation Kit (Millipore, Billerica, MA, USA) according to manufacturer’s protocol. Briefly, U87 cells were first lysed with the complete RIP lysis buffer. Next, the total cell lysate (100 μl) from each group was incubated with RIP buffer containing mouse IgG (normal control) or mouse anti-Ago2 antibody (Millipore, USA)-conjugated magnetic beads. Subsequently, cells were incubated with proteinase K for protein digestion, followed by collection of immunoprecipitated RNA. A NanoDrop spectrophotometer (Thermo Scientific, USA) was then used to quantify RNA content, followed by reverse transcription of the purified RNA into cDNA and determination of miR-128-3p and FOXD3-AS1-AS1 levels using qPCR.

### Dual luciferase assay

Bioinformatics databases (Starbase and Pictar) were employed to predict the binding sites of miR-128-3p on WEE1 and FOXD3-AS1. Next, we cloned and inserted the wild-type (WT) binding site and the flanking sequences (~300 bp) in HindIII and SpeI sites into the pMIR-REPORT Luciferase vector, which were then referred to as WEE1-WT and FOXD3-AS1-WT, respectively. Point mutations were introduced at the binding sites using a Phusion Site-Directed Mutagenesis Kit (Thermo Fisher Scientific, USA) according to the manufacturer’s instructions. Notably, the mutant forms were referred to as FOXD3-AS1-Mut and WEE1-Mut, respectively.

Furthermore, lipofectamine 2000 (Invitrogen) was used to transfect specific reagents into U87 cells after inoculation in 6-well plates for 48 h. The luciferase activity was then measured using the Dual Luciferase reporter 1000 Assay System (Promega, Madison, WI, USA). Notably, the activity of Renilla was adopted for normalization.

### Statistical analyses

All statistical analyses were performed using SPSS17.0 (SPSS, Chicago, IL, USA) software. All data were expressed as mean ± SD for the three separate assays. Two-tailed student’s t-test was used to compare differences between two groups, whereas analysis of variance (ANOVA) was used to compare differences among multiple groups. P < 0.05 was regarded as statistically significant.

## Results

This study explored the role of FOXD3-AS1 in the survival and TMZ resistance of GBM cells, and elucidated its underlying molecular mechanism. Functional experiments revealed that FOXD3-AS1 promoted WEE1 expression through sponging miR-128-3p to increase GBM cell survival and TMZ resistance, suggesting that FOXD3-AS1 might be a potential therapeutic target for GBM treatment.

### FOXD3-AS1 is upregulated in GBM and promotes survival of GBM cells

**Figure 1.**
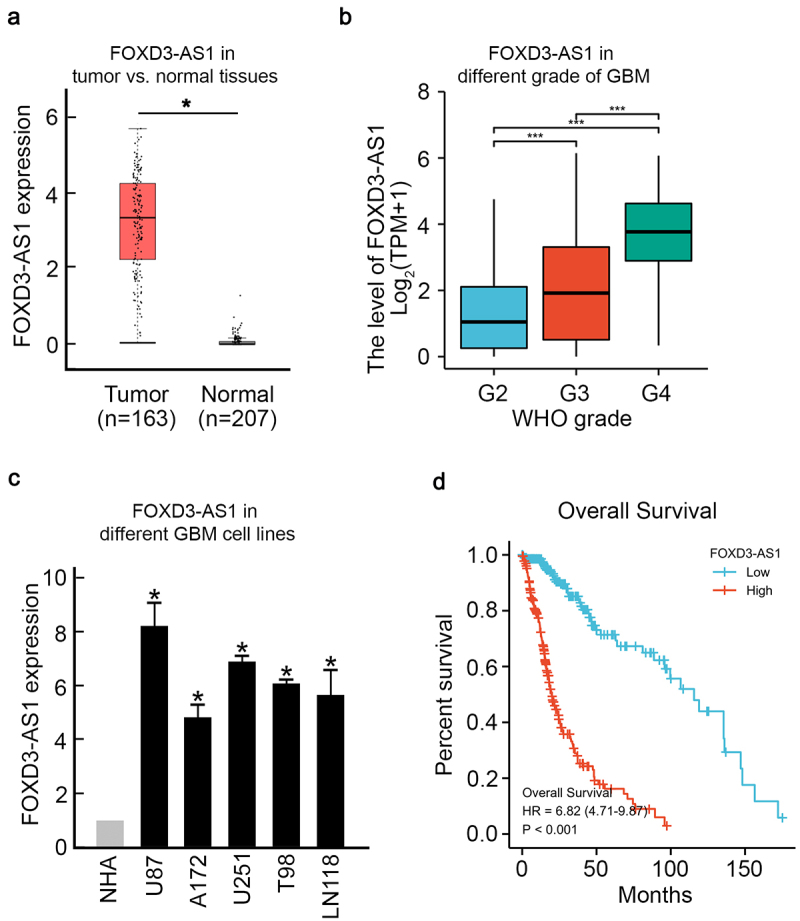
Elevated FOXD3-AS1 was observed in GBM and is correlated with poor prognosis.

To evaluate the effect of FOXD3-AS1 on GBM, we first analyzed patients’ data retrieved from The Cancer Genome Atlas (TCGA) database. Results showed a significant upregulation of FOXD3-AS1 in GBM cancer samples compared to healthy samples (Figure 1a). And Higher level of FOXD3-AS1 was found in higher WHO grade of GBM samples (Figure 1b). Consistently, FOXD3-AS1 upregulation was observed in five different GBM cell lines, including U87, A172, U251, T98, and LN118 cells, compared to NHA cells (normal human astrocytes, Figure 1c). Moreover, Kaplan–Meier analysis showed that patients with higher levels of FOXD3-AS1 had a significantly worse prognosis (Figure 1d). Altogether, these results suggest that elevated FOXD3-AS1 plays a vital role in GBM.

**(A)** According to the TCGA dataset, FOXD3-AS1 expression was upregulated in GBM patient tissue samples (*p < 0.05). **(B)** According to the TCGA dataset, level of FOXD3-AS1 was remarkably higher in higher WHO grade of GBM patient. (*p < 0.05) **(C)** qRT-PCR results showed that FOXD3-AS1 expression was increased in different GBM cell lines compared to normal NHA cells (N = 3, *p < 0.05). **(D)** Kaplan–Meier analysis indicated that a higher level of FOXD3-AS1 indicated worse prognosis for GBM patients.

**Figure 2.**
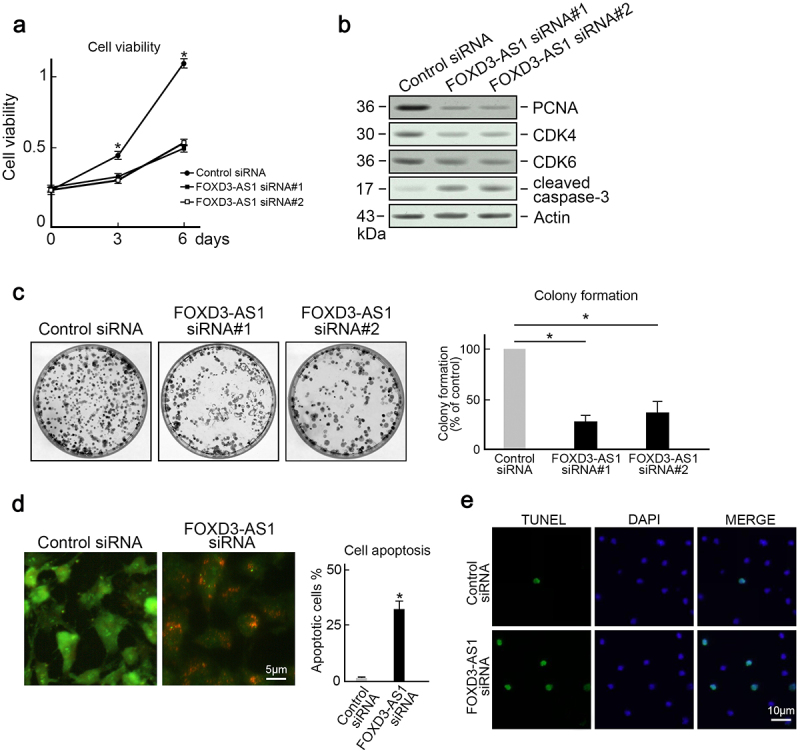
**Depletion of FOXD3-AS1 inhibited proliferation and induced apoptosis in U87 cells**. (a) Depletion of FOXD3-AS1 reduced growth of U87 cells. U87 cells transfected with FOXD3-AS1 siRNA#1 or siRNA#2 or control siRNA were seeded into 96-well plates. The MTT assay was then used to evaluate cell viability at day 0, day 3, and day 6 after seeding (N = 3, *p < 0.05). (b) Control or FOXD3-AS1 siRNA#1 or siRNA#2 was transfected into U87 cells, followed by immunoblotting to examine levels of PCNA, CDK4, CDK6, and cleaved caspase-3 in U87 cells. Actin served as the loading control. (c) The colony formation assay indicated that FOXD3-AS1 deletion suppressed cell survival in U87 cells (N = 3, *p < 0.05). (d) AO/EB staining showed that FOXD3-AS1 deletion promoted U87 cells apoptosis (N = 3, *p < 0.05). (e) TUNLE staining results revealed that FOXD3-AS1 deletion promoted U87 cells apoptosis (N = 3).

To investigate the biological functions of FOXD3-AS1 in GMB, U87 cells and U251 cells (which harbored the highest FOXD3-AS1 expression) were utilized as the in vitro model. Results indicated that the downregulation of FOXD3-AS1 suppressed the growth of U87 cells and U251 cells (Figure 2a, Figure S1). Several factors that accelerate cell cycle, such as CDK4, PCNA, and CDK6, were downregulated in FOXD3-AS1-deleted U87 cells (Figure 2b). In addition, cleaved caspase-3 expression was elevated after FOXD3-AS1 was depleted (Figure 2b). The clone forming experiment also revealed that less colonies were formed after FOXD3-AS1 depletion (Figure 2c). Moreover, AO/EB staining revealed that depletion of FOXD3-AS1 increased the proportion of apoptotic cells in U87 cells (Figure 2d). This observation was further confirmed by TUNEL staining (Figure 2e). These results suggest that FOXD3-AS1 promoted survival of GBM cells.

### FOXD3-AS1 contributes to TMZ resistance in GBM cells

**Figure 3.**
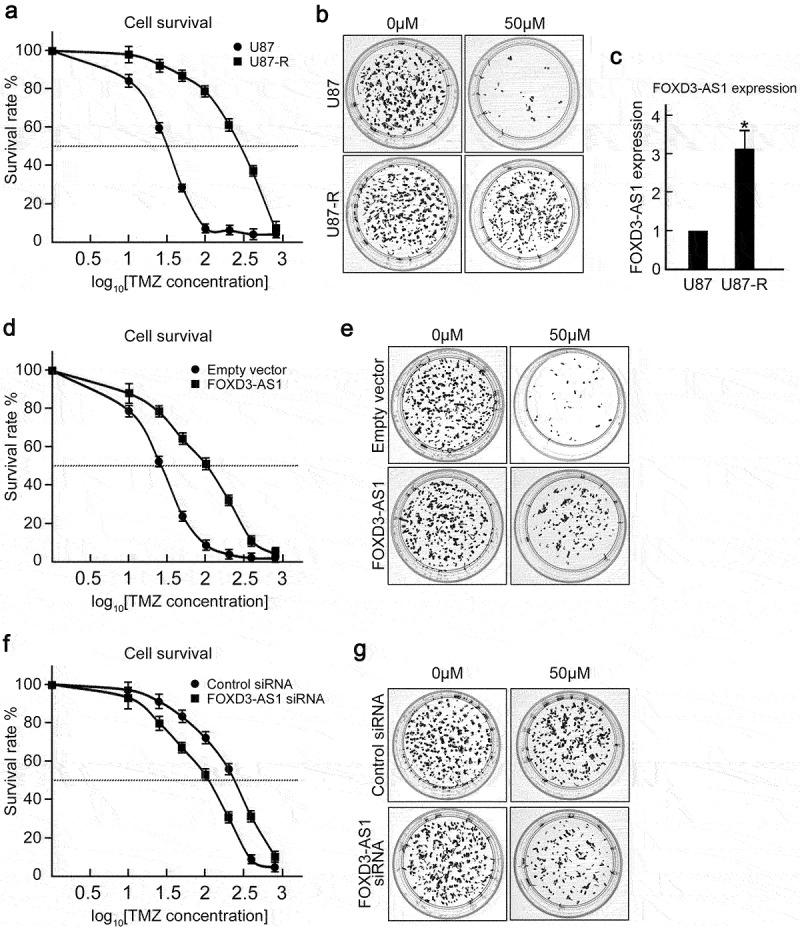
FOXD3-AS1 contributed to TMZ resistance in U87 cells.

Considering that FOXD3-AS1 was critical for survival of GBM cells, we further explored the effect of FOXD3-AS1 deletion on enhancing TMZ sensitivity of GBM cells. First, we established TMZ resistant U87 cells (U87-R) through treating U87 cells with gradient TMZ concentrations. The U87-R cells exhibited enhanced tolerance to TMZ, with increased IC_50_ from 32.35 μM to 287.33 μM (Figure 3a). Colony formation under 50 μM TMZ treatment confirmed that quite a few colonies were counted in U87 cells, whereas U87-R cells could form almost as many colonies as untreated groups (Figure 3b). As expected, it was found that U87-R cells harbored significantly higher FOXD3-AS1 expression compared to parental U87 cells (Figure 3c). Overexpression of FOXD3-AS1 caused TMZ sensitive U87 and U251 cells to be more tolerant to TMZ treatment, with increased IC_50_ from 32.35 μM to 102.52 μM (U87 cells) and from 40.74 μM to 153.43 μM (Figure 3d-E, Figure. S2). Interestingly, we found that depletion of FOXD3-AS1 could sensitize U87-R cells to TMZ treatment compared to control siRNA transfected cells (figure 3f-G). These results suggest that FOXD3-AS1 contributed to TMZ resistance in GBM cells.

**(A)** Parental U87 cells and TMZ resistant U87-R cells (5000/well) were seeded into the 96-well plates, followed by six days of 0, 10, 25, 50, 100, 200, 400, and 800 μM TMZ treatment. MTT assay was then conducted to assess cell survival (N = 3). **(B)** Clone forming assay: U87 and U87-R cell lines were seeded into the 6-cm dishes (800 cells per dish), followed by TMZ treatment at 0 and 50 μM. Representative plates are shown. N = 3. **(C)** qRT-PCR results showed that FOXD3-AS1 expression was increased in U87-R cells compared to parental U87 cells (N = 3, *p < 0.05). **(D)** Over-expression of FOXD3-AS1 increased IC_50_ of TMZ treatment in U87 cells. N = 3. **(E)** The colony formation assay indicated that U87 cells overexpressing FOXD3-AS1 were more tolerant to TMZ treatment. Representative plates are shown. N = 3. **(F)** Depletion of FOXD3-AS1 decreased IC_50_ of TMZ treatment in U87-R cells (N = 3). **(G)** The colony formation assay also showed that FOXD3-AS1-depleted U87-R cells were more sensitive to TMZ treatment. Representative plates are shown. N = 3.

### FOXD3-AS1 sponges miR-128-3p to promote WEE1 expression

**Figure 4.**
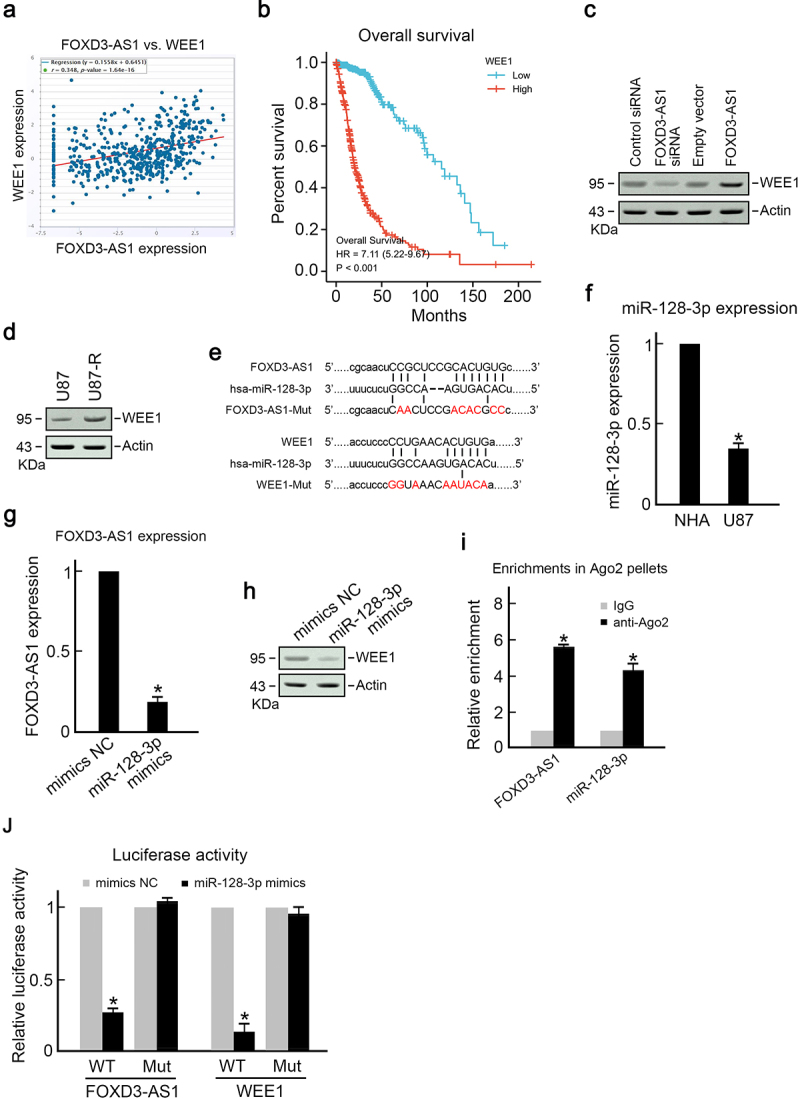
FOXD3-AS1 promoted WEE1 expression by sponging miR-128-3p.

To elucidate the mechanism through which FOXD3-AS1 promotes survival of GBM cells and TMZ resistance, we first analyzed the TCGA dataset using Starbase 3.0 project and found that WEE1, an important cell-cycle regulator, was positively correlated with FOXD3-AS1 expression (Figure 4a). Results showed that GBM patients with higher levels of WEE1 had poorer prognosis (Figure 4b), which is similar to FOXD3-AS1. Downregulation of FOXD3-AS1 suppressed WEE1 expression, whereas overexpression of FOXD3-AS1 promoted WEE1 expression (Figure 4c). Importantly, it was found that the expression of WEE1 in TMZ resistant U87-R cells was significantly higher than that in parental TMZ sensitive U87 cells (Figure 4d). Moreover, bioinformatics analysis using Starbase and Pictar revealed that the possible miR-128-3p binding sites were on 3’-UTR of WEE1 and FOXD3-AS1 (Figure 4e), which suggested that FOXD3-AS1 may enhance WEE1 expression through the sponge of miR-128-3p. Therefore, we analyzed miR-128-3p expression in U87 cells. As expected, U87 cells exhibited significantly decreased miR-128-3p expression compared to healthy NHA cells (figure 4f). Overexpression of miR-128-3p downregulated FOXD3-AS1 and WEE1 in U87 cells (Figure 4g, H). The RIP assay showed presence of miR-128-3p and FOXD3-AS1 in the identical RISC complex. As shown in Figure 4i, the enrichments of miR-128-3p and FOXD3-AS1 were significantly higher in precipitated Ago2 pellets compared to IgG pellets (Figure 4i). Meanwhile, the dual-luciferase assay demonstrated that miR-128-3p mimics significantly decreased the luciferase activity of FOXD3-AS1-WT cells compared to that of mutant groups (Figure 4j). Similarly, miR-128-3p mimics reduced the luciferase activity of WEE1-WT cells, but did not alter the luciferase activity in WEE-Mut groups (Figure 4j). Collectively, these results suggest that FOXD3-AS1 promoted the WEE1 level through the sponge of miR-128-3p.

**(A)** Positive correlation of FOXD3-AS1 and WEE1 in GBM patients. Data from TCGA database. **(B)** Kaplan–Meier analysis results showed that higher levels of WEE1 were associated with poorer prognosis of GBM patients. **(C)** After transfection using specific reagents, immunoblotting was conducted to determine WEE1 expression in U87 cells, with actin as a loading reference. **(D)** The WEE1 level was elevated in U87-R cells compared to the parental U87 cells, with actin as a loading reference. **(E)** Sketch map showing possible binding sites of miR-128-3p in WEE1 and FOXD3-AS1.**(F)** MiR-128-3p level was elevated in U87 cells compared to healthy NHA cells (N = 3, *p < 0.05). **(G)** Overexpression of miR-128-3p reduced FOXD3-AS1 level in U87 cells (N = 3, *p < 0.05). **(H)** Overexpression of miR-128-3p suppressed WEE1 expression in U87 cells. Notably, actin served as the loading control. **(I)** Relative abundances of miR-128-3p and FOXD3-AS1 in the RISC complex detected using the RIP assay with the use of anti-Ago2 antibody (N = 3, *p < 0.05). **(J)** Dual-luciferase reporter assay. Specific reagents were used to transfect U87 cells for 48 h, and then the relative luciferase activities were measured using the Dual-Luciferase Reporter assay system (Promega) (N = 3, *p < 0.05).

### FOXD3-AS1 promotes TMZ resistance in GBM through upregulation of WEE1

**Figure 5.**
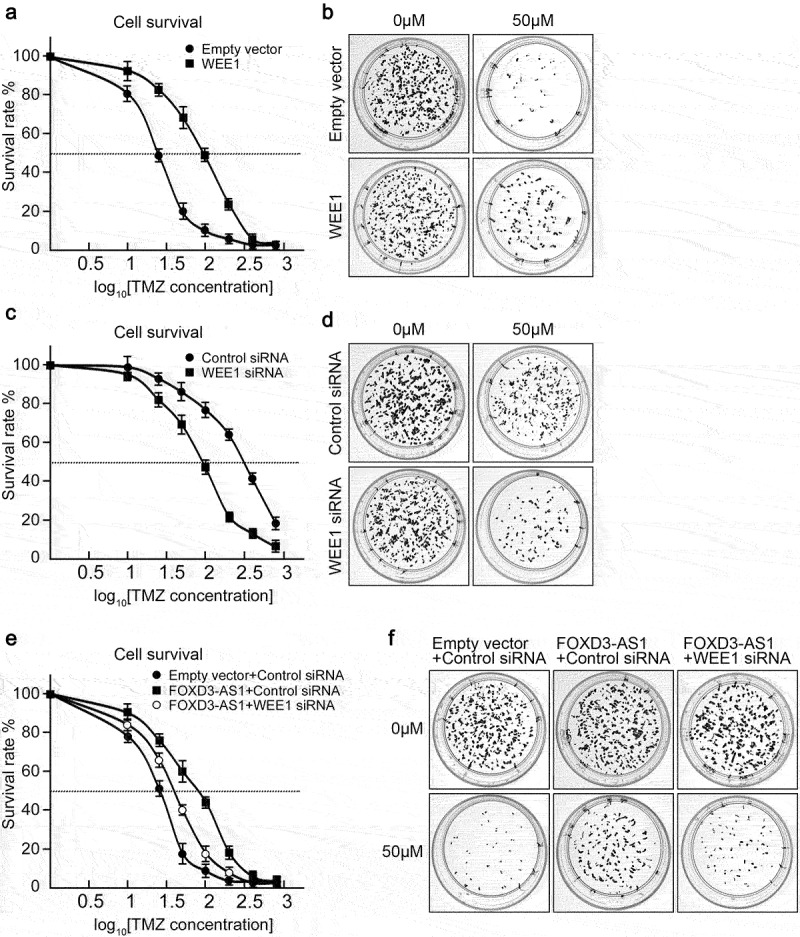
FOXD3-AS1 promoted TMZ resistance through upregulation of WEE1 in U87 cells.

Given that FOXD3-AS1 facilitated WEE1 expression in U87 cells, we further explored whether FOXD3-AS1 promoted TMZ resistance in GBM through WEE1. WEE1 inhibition could arrest cell cycle at G2/M [22], thus we analyzed the cell cycle upon FOXD3-AS1 knockdown. We found that knockdown of FOXD3-AS1 increased the populations of G2/M, but decreased the populations of S phase cells (Figure S3), indicating that knockdown of FOXD3-AS1, which results in suppression of WEE1 expression, induced cells accumulation in the G2/M phase. Later, U87 cells overexpressing WEE1 exhibited higher IC_50_ compared to control cells, suggesting that they were more tolerant to TMZ treatment (Figure 5a, b). Knockdown of WEE1 could sensitize U87-R cells to TMZ treatment (Figure 5c, d). Considering that the above results have shown that overexpression of FOXD3-AS1 increased TMZ tolerance in U87 cells, we further demonstrated that knockdown of WEE1 could sensitize FOXD3-AS1-overexpressed U87 cells to TMZ treatment (Figure 5e, f). These results indicate that FOXD3-AS1 promoted TMZ resistance in GBM through upregulation of WEE1.

**(A)** Overexpression of WEE1 increased IC_50_ of TMZ treatment in U87 cells. N = 3. **(B)** The colony formation assay showed that U87 cells with overexpression of WEE1 were more tolerant to TMZ treatment. Representative plates are shown. N = 3. **(C)** Depletion of WEE1 decreased the IC_50_ of TMZ treatment in U87-R cells. N = 3. **(D)** The colony formation assay also indicated that U87-R cells with depleted WEE1 were more sensitive to TMZ treatment. Representative plates are shown. N = 3. **(E)** Depletion of WEE1 could reverse the TMZ tolerance of U87 cells induced by overexpression of FOXD3-AS1. After transfection with the indicated reagents, and exposure to 0, 10, 25, 50, 100, 200, 400, and 800 μM TMZ for six days, the survival rate of U87 cells was assessed by the MTT assay. N = 3. **(F)** Colony formation assay. U87 cells were subjected to specific reagent transfection and simultaneous TMZ exposure at 0 and 50 μM. Representative plates are shown. N = 3.

## Discussion

TMZ-based chemotherapy is one of the standard treatments for glioblastoma (GBM), which is a highly aggressive brain tumor [5]. However, studies have shown that majority of GBM patients suffer from tumor recurrence after initial treatment and develop TMZ resistance [23]. Thus, there is an urgent need to elucidate the mechanisms through which GBM cells enhance TMZ tolerance.

FOXD3-AS1 has been shown to dominate an oncogenic role in different cancer types. For example, the upregulation of FOXD3-AS1 enhances the development of NSCLC [24], whereas inhibition of FOXD3-AS1 restrains the aggressiveness of thyroid cancer cells through inactivation of the TGF-β1/Smads pathway [25]. FOXD3-AS1 also promotes chemoresistance of NSCLC through a direct effect on the miR-127-3p/MDM2 axis [26]. In addition, a recent study revealed that FOXD3-AS1 is involved in anti-estrogen resistance in breast cancer [27]. These findings suggest that FOXD3-AS1 may play a critical role in drug resistance in cancer. Results obtained in this study showed that elevated expression of FOXD3-AS1 in GBM cells was significantly correlated with poor prognosis in GBM patients and contributed to TMZ resistance. It was also found that FOXD3-AS1 expression was higher in GBM cells than in normal cells, and the expression was even significantly increased in TMZ-tolerant GBM cells. Further experiments revealed that FOXD3-AS1 promoted survival of GBM cells, whereas FOXD3-AS1 deletion enhanced the sensitivity of U87-R cells to TMZ. This finding suggests that FOXD3-AS1 has an important function in modulating the tolerance of GBM cells to TMZ.

WEE1 is a key kinase in maintaining G2/M checkpoint arrest for pre-mitotic DNA repair [22]. WEE1 inactivates CDK1 through inhibitory phosphorylation at Ser15, thereby preventing mitosis entry upon cell DNA damage [28]. Several studies have reported that WEE1 is highly expressed in various cancer types [29,30]. Herein, we found that the level of WEE1 in GBM cells was higher than that in normal cells, and even much higher in TMZ resistant GBM cells. This phenotype of WEE1 upregulation was attributed to the elevated expression of FOXD3-AS1. Specifically, FOXD3-AS1 acted as a ceRNA to sponge miR-128-3p, and subsequently promoted WEE1 expression. Previous studies have proved that cancer cells expressing WEE1 rely on an intact G2/M checkpoint for survival and mitosis [22]. Given that activation of WEE1 contributes to chemoresistance in different cancers [31,32], inhibition of WEE1 may be a possible anti-tumor treatment [33,34]. This study has also shown that overexpression of WEE1 could sensitize GBM cells to be more tolerant to TMZ treatment, whereas depletion of WEE1 could sensitize TMZ-resistant cells to TMZ treatment. Importantly, depletion of WEE1 could reverse the resistant phenotype in FOXD3-AS1 overexpressed cells. Collectively, these findings suggest that WEE1 is vital for TMZ resistance and FOXD3-AS1 promotes TMZ resistance in GBM cells through upregulation of WEE1.

## Conclusions

In conclusion, this study has revealed an important role of FOXD3-AS1 in GBM. Elevated expression of FOXD3-AS1 was found in GBM patients, which was associated with poor prognosis. Results have also shown that FOXD3-AS1 functions as a ceRNA to promote WEE1 expression by competitively binding to miR-128-3p, thereby promoting GBM cell survival and conferring TMZ resistance in GBM cells.

## Supplementary Material

Supplemental Material

## Data Availability

The data that support the findings of this study are available from the corresponding author upon reasonable request.
