# Supplementary figures and images for: Oncogenic Forkhead box D3 antisense RNA 1 promotes cell survival and confers temozolomide resistance in glioblastoma cells through the miR-128-3p/WEE1 G2 checkpoint kinase axis
Source: Bioengineered. 2022 Feb 22;13(3):6012–23. doi: 10.1080/21655979.2022.2042133 (PMC8974031; doi:10.1080/21655979.2022.2042133)

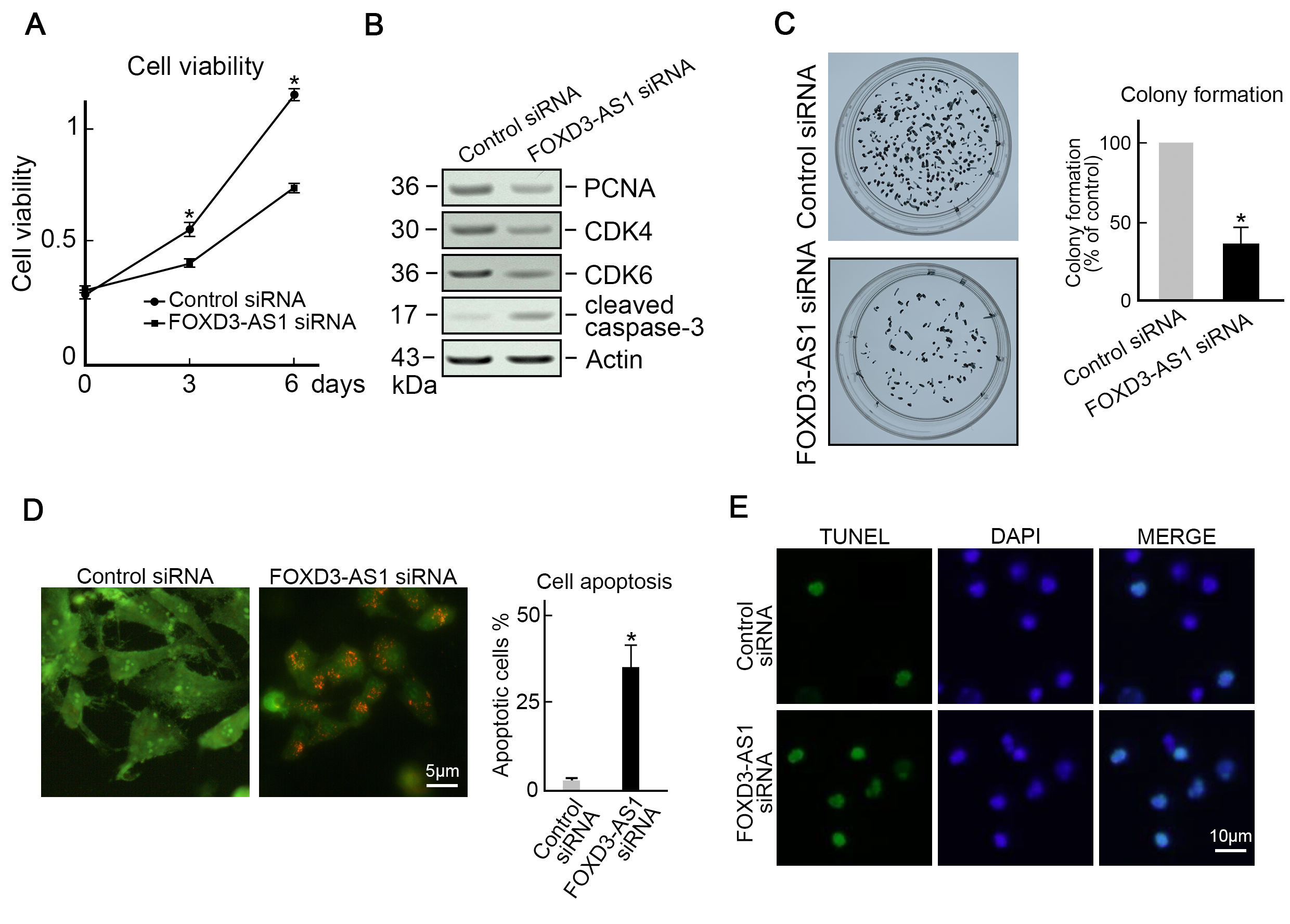

Supplement: Supplemental Material [file KBIE_A_2042133_SM5053.zip › supplementary/FigS1.jpg]

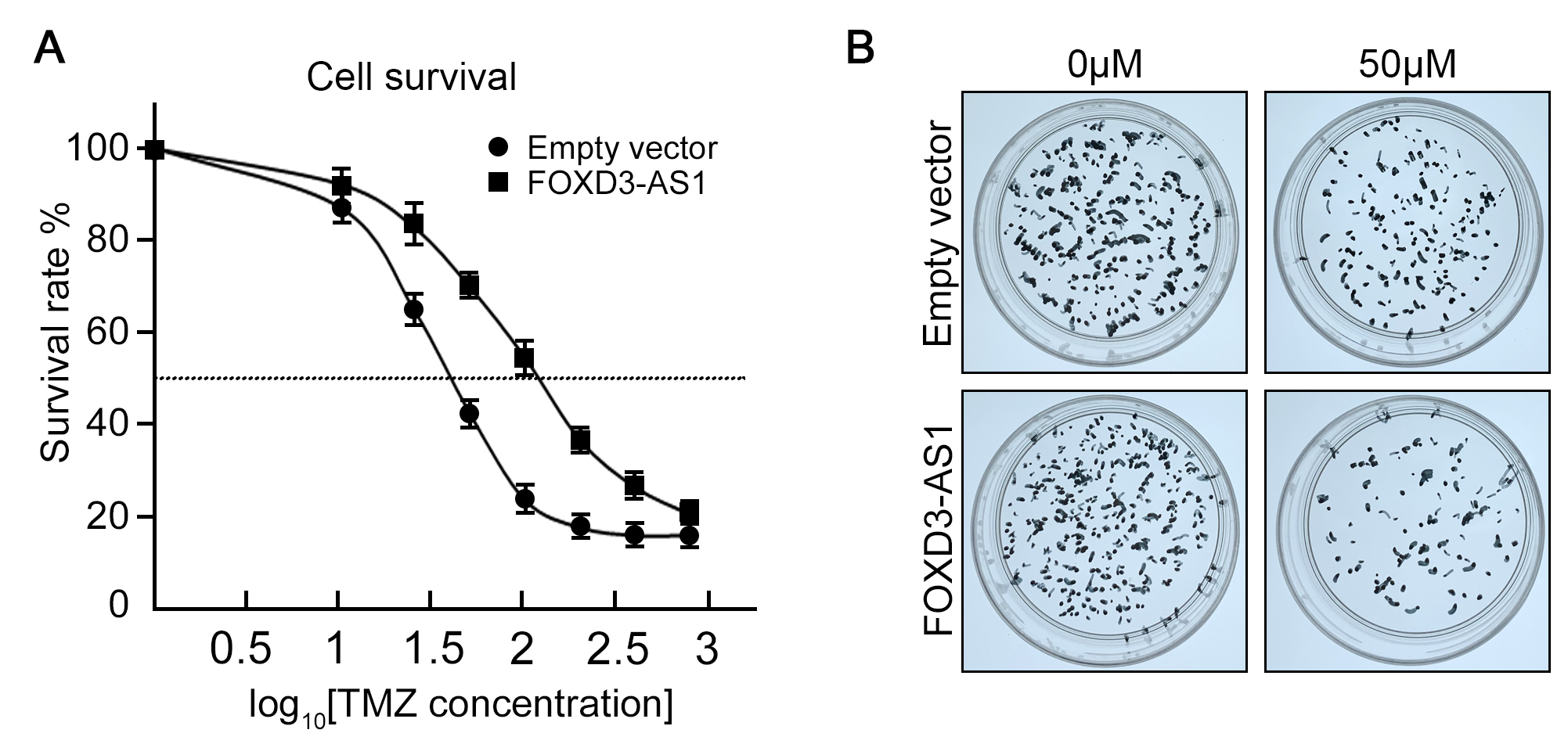

Supplement: Supplemental Material [file KBIE_A_2042133_SM5053.zip › supplementary/FigS2.jpg]

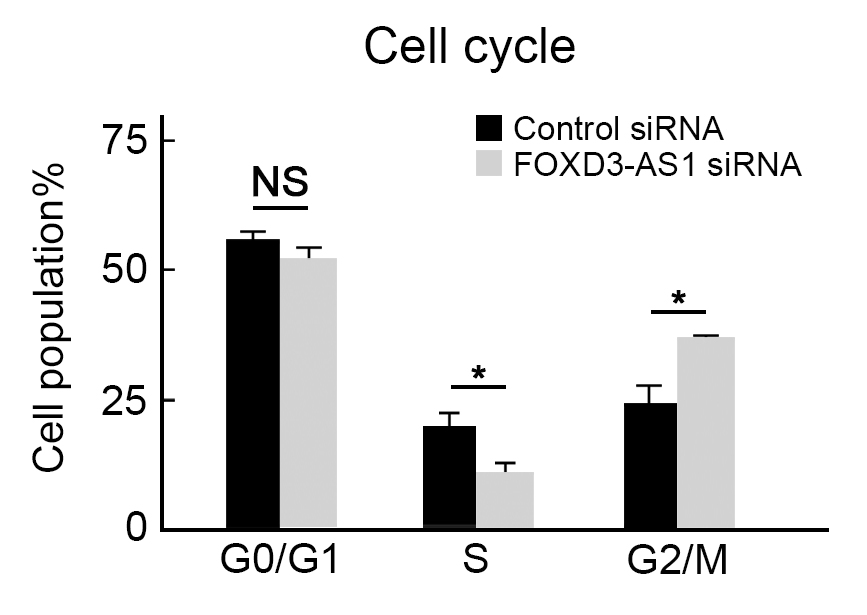

Supplement: Supplemental Material [file KBIE_A_2042133_SM5053.zip › supplementary/FigS3.jpg]
